# Influencing factors and mechanism of Cr(VI) reduction by facultative anaerobic *Exiguobacterium* sp. PY14

**DOI:** 10.3389/fmicb.2023.1242410

**Published:** 2023-08-10

**Authors:** Yunhong Huang, Jie Tang, Bei Zhang, Zhong-Er Long, Haiyan Ni, Xueqin Fu, Long Zou

**Affiliations:** ^1^Nanchang Key Laboratory of Microbial Resources Exploitation and Utilization from Poyang Lake Wetland, College of Life Sciences, Jiangxi Normal University, Nanchang, China; ^2^College of Art and Design, Jiangxi Institute of Fashion Technology, Nanchang, China

**Keywords:** Cr(VI) reduction, *Exiguobacterium*, heavy metal, chromate reductase, organo-Cr(III)

## Abstract

Microbial reduction is an effective way to deal with hexavalent chromium [Cr(VI)] contamination in the environment, which can significantly mitigate the biotoxicity and migration of this pollutant. The present study investigated the influence of environmental factors on aqueous Cr(VI) removal by a newly isolated facultative anaerobic bacterium, *Exiguobacterium* sp. PY14, and revealed the reduction mechanism. This strain with a minimum inhibitory concentration of 400 mg/L showed the strongest Cr(VI) removal capacity at pH 8.0 because of its basophilic nature, which was obviously depressed by increasing the Cr(VI) initial concentration under both aerobic and anaerobic conditions. In contrast, the removal rate constant for 50 mg/L of Cr(VI) under anaerobic conditions (1.82 × 10^−2^ h^−1^) was 3.3 times that under aerobic conditions. The co-existence of Fe(III) and Cu(II) significantly promoted the removal of Cr(VI), while Ag(I), Pb(II), Zn(II), and Cd(II) inhibited it. Electron-shuttling organics such as riboflavin, humic acid, and anthraquinone-2,6-disulfonate promoted the Cr(VI) removal to varying degrees, and the enhancement was more significant under anaerobic conditions. The removal of aqueous Cr(VI) by strain PY14 was demonstrated to be due to cytoplasmic rather than extracellular reduction by analyzing the contributions of different cell components, and the end products existed in the aqueous solution in the form of organo-Cr(III) complexes. Several possible genes involved in Cr(VI) metabolism, including *chrR* and *chrA* that encode well-known Chr family proteins responsible for chromate reduction and transport, respectively, were identified in the genome of PY14, which further clarified the Cr(VI) reduction pathway of this strain. The research progress in the influence of crucial environmental factors and biological reduction mechanisms will help promote the potential application of *Exiguobacterium* sp. PY14 with high adaptability to environmental stress in Cr(VI) removal in the actual environment.

## 1. Introduction

Chromium (Cr) has a variety of significant industrial applications in textile dyes and mordants, electroplating, alloying, pigments, leather tanning, refractories, and ceramic glazes (Xia et al., 2020; Ukhurebor et al., 2021). Nevertheless, the accompanying Cr accumulation in the environment and its ecological risks have become important global concerns. The prevalent forms of Cr in the natural environment are trivalent Cr(III) and hexavalent Cr(VI) (Kotas and Stasicka, 2000; Rahman and Thomas, 2021), of which Cr(VI) is more toxic, migratory, and bioavailable (Jiang et al., 2019). Cr(VI) usually exists in several highly soluble forms of oxyanions varying with the pH of the aqueous media (Ukhurebor et al., 2021; Zheng et al., 2021), while Cr(III) behaves as a cation and can form precipitates such as Cr(OH)_3_ (Rahman and Thomas, 2021). Moreover, Cr(VI), with its high oxidizing potential, exhibits strong cytotoxicity and DNA damage (DesMarais and Costa, 2019) and has been classified as a group 1 carcinogen by the International Agency for Research on Cancer (IARC, 1980). Therefore, the reduction of Cr(VI) to Cr(III) is recognized as an effective route to remediate Cr pollution in the soil and water ecosystems.

Compared with a conventional chemical route, which usually uses a large number of chemicals and produces enormous toxic sludge (Peng and Guo, 2020), microbial remediation of Cr(VI) is characterized as a fast, economical, environmental friendly, and low-energy consumption process, thus being considered a promising disposal strategy to counteract Cr(VI) pollution (Sharma et al., 2022). Numerous bacteria, both Gram-negative and Gram-positive, have shown their ability to remove Cr(VI) through diverse mechanisms, including biosorption on the cell surface, bioaccumulation inside the cell, and biotransformation by the involvement of specific chromate reductases or other non-specific ones (Jobby et al., 2018). More particularly, abundant bacteria such as *Bacillus* sp. (Zhu et al., 2019; Gu et al., 2022), *Pseudomonas* sp. (Yu et al., 2016; Kang et al., 2017), *Alishewanella* sp. (Xia et al., 2018), *Leucobacter* sp. (Zhu et al., 2008), *Rhodococcus* sp. (Banerjee et al., 2017), *Shewanella* sp. (Bencheikh-Latmani et al., 2005; Parker et al., 2011), and *Exiguobacterium* sp. (Alam and Malik, 2008; Mohapatra et al., 2017; Das et al., 2021) have been well documented as being capable of reducing Cr(VI) either intracellularly or extracellularly. However, their Cr(VI)-reducing mechanisms differ greatly, which determines the removal efficiency and application scenarios of these bacteria.

Among them, the genus *Exiguobacterium* is composed of Gram-positive facultative anaerobes with variable morphologies ranging from small rods to cocci, which have been repeatedly isolated or molecularly detected from diverse habitats with a broad range of temperature, pH, and salt ion concentrations (Vishnivetskaya et al., 2009; Kasana and Pandey, 2018). In addition, some strains of this genus have been shown to have plant growth-promoting attributes (Selvakumar et al., 2010; Bharti et al., 2013). These merits endow the genus *Exiguobacterium* with a strong competitive edge in environmental remediation. To our knowledge, the removal capacities and effective behaviors of several strains of *Exiguobacterium* for Cr(VI) remediation have been reported under aerobic conditions (Alam and Malik, 2008; Okeke, 2008; Mohapatra et al., 2017; Das et al., 2021), but there are still knowledge gaps in key influencing factors under both aerobic and anaerobic environments, as well as their biological reduction mechanisms. In our previous study, an alkaliphilic and salt-tolerant strain of *Exiguobacterium* sp. PY14 was isolated from the wetland soil of Poyang Lake (the largest freshwater lake in China), which could decolorize azo dyes and extracellularly reduce insoluble iron-containing minerals (Tang et al., 2023). Herein, the reductive removal of aqueous Cr(VI) by this strain and the influencing factors were studied. Moreover, the reduction mechanism was discussed based on the distribution and speciation of reduction products, the contribution of different cell components, and potential genes related to Cr(VI) metabolism. This study would provide insights into the application of the genus *Exiguobacterium* in the remediation of Cr(VI) contamination.

## 2. Materials and methods

### 2.1. Strain and culture

Strain PY14 used in this study was identified as being 98% similar to *E. mexicanum* 8N (GenBank assembly accession: GCA_025234665.1) based on 16S rRNA gene sequencing (Tang et al., 2023). This strain was cultured in 250-mL Erlenmeyer flasks with Luria-Bartani (LB) broth (tryptone, 10 g/L; yeast extract, 5 g/L; NaCl, 10 g/L; pH 8.5) for a logarithmic growth period as inoculants for follow-up Cr(VI) removal tests.

### 2.2. Cr(VI) removal test

The tests of Cr(VI) removal by PY14 cells were performed in LB broth medium only containing potassium dichromate (K_2_CrO_4_) as the form of Cr(VI) unless otherwise stated, and the bacterial cultures were inoculated in the fresh medium by 1% volume. The aerobic and anaerobic tests were carried out in 250-mL Erlenmeyer flasks and 100-mL serum vials, respectively, with a working volume of 50 mL. To achieve anaerobic conditions, the inoculums in serum vials were sparged with a mixture of N_2_:CO_2_ (80:20) to remove dissolved oxygen and capped with butyl rubber closures. The Erlenmeyer flasks and serum vials were incubated on a rotary shaker (200 rpm) at 37^o^C. During the incubation period, 1 mL aliquots of the cultures were periodically withdrawn using a sterile syringe and used for analysis after being filtered through a sterile aqueous microporous membrane (average pore size of 0.22 μm). The effects of the initial pH of the medium, initial Cr(VI) concentration, co-existing metal ions, and organic electron shuttle were investigated through the single-factor control experiment. The initial pH of the medium was adjusted by adding 0.1 M HCl or NaOH. The initial Cr(VI) concentration was controlled by changing the additional amount of Cr(VI) stock solution (10 g/L). The influences of Pb(II), Ag(I), Cu(II), Fe(III), Zn(II), and Cd(II) were studied by adding corresponding chlorates at a concentration of 20 mg/L. Anthraquinone-2,6-disulfonate (AQDS), riboflavin, and humic acid were selected as typical electron-shuttling organics and used at a concentration of 10 μM. Each experiment was performed in triplicate.

### 2.3. Cell denaturation and fraction preparation

To investigate the mechanism of Cr(VI) reduction by strain PY14, the cells were treated with different denaturation methods. In detail, after culture in LB medium without Cr(VI) for a logarithmic growth period, the cells were collected by centrifugation at 8,000 rpm for 5 min, and the cell-free culture supernatants were stored at 4^o^C for later use. The collected cells were resuspended in an equal volume of fresh LB medium, and the cell suspension was divided into three parts. One part was not further treated and was directly used for the resting cells. Another part was incubated at 80^o^C for 30 min to obtain the heat-killed cells. The last part was treated with 5 mM sodium dodecyl sulfate polyacrylamide (SDS) for 30 min to denature proteins, resulting in SDS-denatured cells (Huang et al., 2021b). The prepared cell-free culture supernatant, resting cells, heat-killed cells, and SDS-denatured cells were used to remove Cr(VI) at an initial concentration of 20 mg/L.

Meanwhile, the cells at the logarithmic growth period were also resuspended in a 0.01-M phosphate buffer solution (PBS, pH = 8.0), and the suspension was divided into four parts. One part was directly used as the resting cells. The other part was used for ultrasonication to obtain the cytoplasmic fraction and cell insoluble fraction after centrifugation at 1,000 rpm for 10 min, and the cell insoluble fraction was resuspended in an equal volume of PBS buffer. The remaining two parts were treated with 5 units of proteinase K and 0.5 mg/mL of lysozyme for 1 h, respectively. All prepared samples were used to reduce Cr(VI) at an initial concentration of 5 mg/L with the addition of 1 mM NADH as electron donors.

### 2.4. Analysis

The density of bacterial cells was detected using an ultraviolet-visible (UV-vis) spectrophotometer (UV-9000S, Shanghai Metash Instruments Co., Ltd., China) at a wavelength of 600 nm. The aqueous Cr(VI) concentration was analyzed using the 1,5-diphenylcarbazide (DPC) UV-vis spectrophotometric method, and K_2_CrO_4_ was used as a standard of known Cr(VI) concentration (Watts et al., 2015). After acid digestion, the total Cr in the cell precipitation was determined using an atomic absorption spectrophotometer (SP-3530AA, Shanghai Spectral Instruments Co., Ltd., China) (Chen et al., 2021). The pH value was measured using a meter (FE28, METTLER TOLEDO, Switzerland). The bacterial morphology and distribution of Cr element on cell precipitates were characterized using scanning electron microscopy (SEM, CLARA, TESCAN, Czech) equipped with energy dispersive spectrometry (EDS). The chemical valence of the Cr element was analyzed using x-ray photoelectron spectroscopy (XPS, AXIS SUPRA, Kratos, UK). The product species of Cr generated in the culture supernatant was examined using x-band electron paramagnetic resonance (EPR, EMXplus, Bruker, Germany) according to previous literature (Puzon et al., 2005).

### 2.5. Genomic DNA sequencing, assembly, and annotation

The draft genome sequencing of strain PY14 was completed by Shanghai Majorbio Bio-pharm Technology Co., Ltd. Genomic DNA was extracted from the collected cells of logarithmic growth using the Genomic DNA Purification Kit (Thermo Scientific™). The harvested DNA was detected by agarose gel electrophoresis and quantified by a Quantus Fluorometer. The library was constructed and sequenced using the Illumina Hiseq × 10 platform, and the pair-end reads were assembled using SOAPdenovo software (Version 2.05). GeneMarkS software (Version 4.3) was used to predict the coding sequence (CDS). The predicted CDSs were translated and used to search against the NR (Non-Redundant Protein Sequence), KEGG (Kyoto Encyclopedia of Genes and Genomes), COG (Clusters of Orthologous Groups), and Swiss-Prot databases. The annotated draft genome has been submitted to GenBank with accession JASWER000000000, and the version described in this article is version JASWER000000000.1. The raw fastq reads were deposited at the NCBI SRA under accession number PRJNA982973.

## 3. Results and discussion

### 3.1. Influence of environmental factors on aqueous Cr(VI) removal

#### 3.1.1. Initial pH

**Figure 1:**
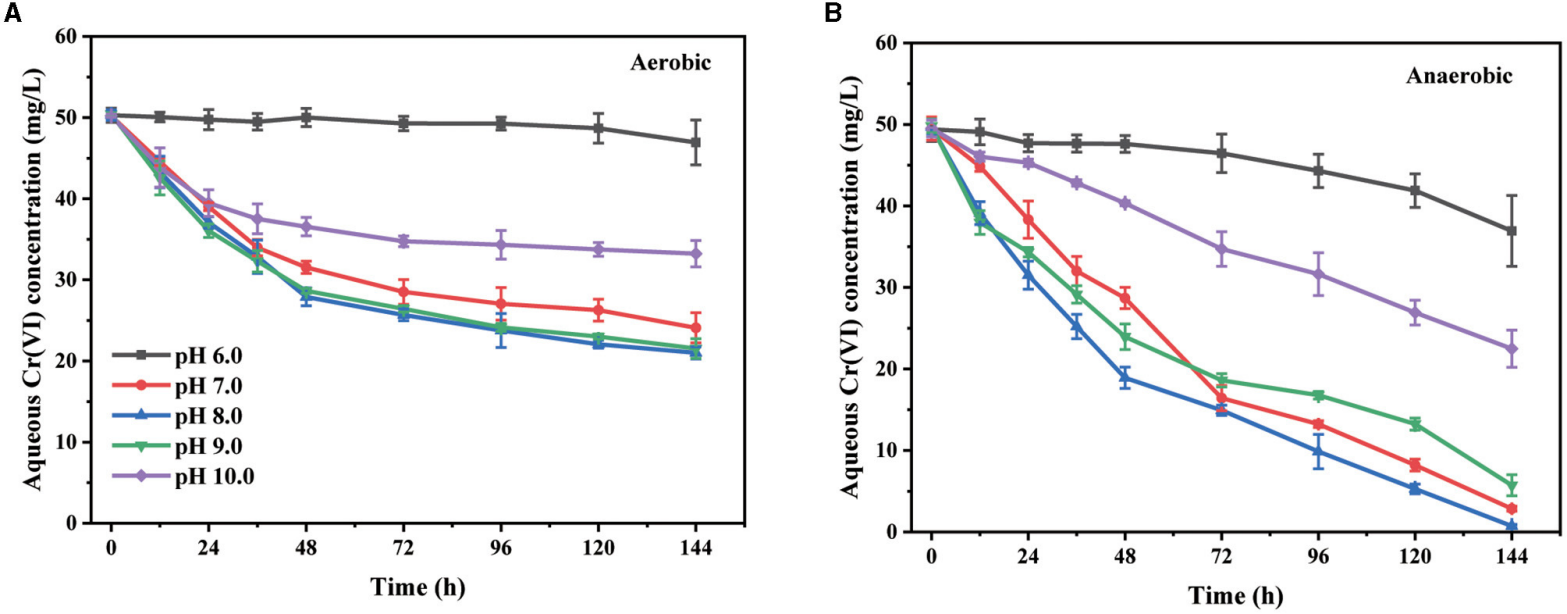
The effect of initial pH value under aerobic **(A)** and anaerobic **(B)** conditions.

The environmental pH is an important factor that affects the existing form of Cr(VI) and the metabolic activity of bacteria. The effect of the initial pH value of LB medium on the Cr(VI) removal by the basophilic *Exiguobacterium* sp. PY14 was first investigated (Figure 1). Under both aerobic and anaerobic conditions, the removal of aqueous Cr(VI) was almost devitalized in the acidic medium, reaching its maximum at an initial pH of 8.0 and then decreasing with a further increase in pH value. The results showed that the strain PY14 exhibited the best removal ability of Cr(VI) in a slightly alkaline environment where the highest density of bacterial cells was obtained (Supplementary Figure S1), regardless of aerobic or anaerobic conditions. After 96 h of reaction, the pH of the medium decreased slightly (Supplementary Figure S1), probably due to the production of a small number of protons during the bacterial metabolism and the relatively weak buffering capacity of the LB medium. This observation was consistent with some previous reports (Mohapatra et al., 2017; Das et al., 2021). Given the fact that the chromate (CrO${\;}_{4}^{2-}$) is the dominant species in an aqueous environment during the tested pH range (Jobby et al., 2018), the observed difference in Cr(VI) removal capacity was suggested to be attributed to the change in bacterial metabolism and protein activity depending on pH value. A high pH value could enhance the electrostatic repulsion between the bacterial surface and the negatively charged form of Cr(VI) (Yaashikaa et al., 2019), which also contributed to the reduced ability at pH over 9.0. In addition, it was obvious that the Cr(VI) removal capacity under anaerobic conditions was much higher than that under aerobic conditions. However, the density of bacterial cells under the former condition was significantly lower than that under the latter condition. Specifically, at the optimum pH, 50 mg/L of Cr(VI) was completely removed within 144 h under anaerobic conditions, while only 58% of that was removed under aerobic conditions. The reasons why the anaerobic condition is better for Cr(VI) removal has been discussed in the following section.

#### 3.1.2. Initial Cr(VI) concentration

**Figure 2:**
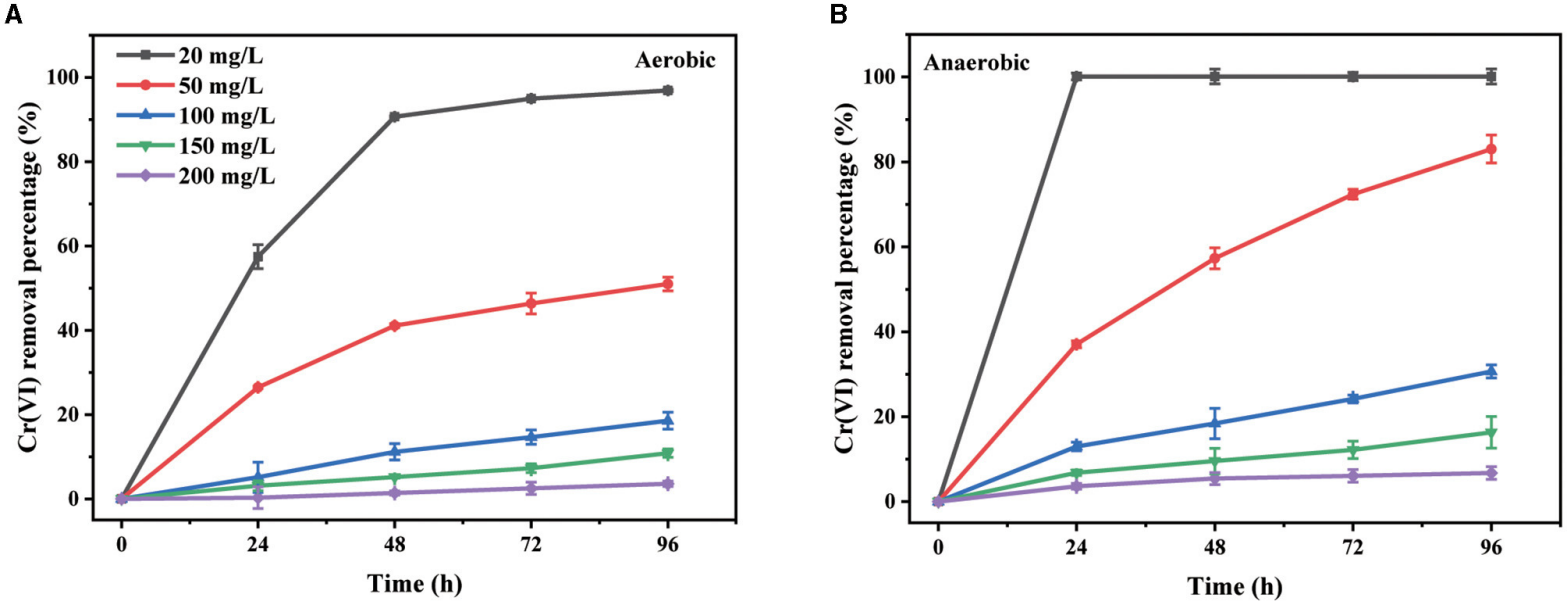
The effect of initial Cr(VI) concentration under aerobic **(A)** and anaerobic **(B)** conditions.

The influence of the initial Cr(VI) concentration from 20 to 200 mg/L, below the detected minimum inhibitory concentration (MIC) of 400 mg/L, on the removal capacity was subsequently analyzed. Under both aerobic and anaerobic conditions (Figure 2), the Cr(VI) removal percentage decreased with an increase in the initial concentration during the 96-h test period. Meanwhile, the values of rate constants derived from the plots of ln(C/C_0_) with time also decreased significantly (Supplementary Figure S2). This was suggested to be attributable to the toxicity of Cr(VI) on this strain (Ramli et al., 2023). The inhibition of Cr(VI) removal ability at the high Cr(VI) concentration was not uncommon (Chen et al., 2021; Das et al., 2021; Huang et al., 2021b). In addition, 20 mg/L of Cr(VI) was removed completely from the culture supernatant within 24 h under anaerobic conditions, while only 57% of that was removed under aerobic conditions. The removal percentage for 50 mg/L of Cr(VI) within 96 h was approximately 82% under anaerobic conditions but only 51% under aerobic conditions. The value of the rate constant for 50 mg/L of Cr(VI) under anaerobic conditions was 1.82 × 10^−2^ h^−1^, which was approximately 3.3-fold of that under aerobic conditions (Supplementary Figure S2). These results further proved that the Cr(VI) removal ability of PY14 under anaerobic conditions was better than that under aerobic conditions. Similar phenomena have been found in some other facultative anaerobic bacteria capable of Cr(VI) reduction, such as *Pannonibacter phragmitetus* LSSE-09 (Xu et al., 2012) and *Sporosarcina saromensis* W5 (Huang et al., 2021b). To the best of our knowledge, the reduction potential of Cr(VI) is approximately 1.33 V (vs. a normal hydrogen electrode), which is higher than that of oxygen. Therefore, the reduction of Cr(VI) is thermodynamically feasible in the presence of oxygen, which explains why PY14 could remove Cr(VI) through bio-reduction under aerobic conditions. Of course, under such conditions, PY14 still used oxygen as an electron acceptor for aerobic respiration, thus competing with Cr(VI) for electrons. However, under anaerobic conditions, the facultative anaerobic bacteria with Cr(VI) reduction ability, including strain PY14, survived with Cr(VI) as a preferred electron acceptor, thus resulting in rapid reductive removal of Cr(VI). Given the appropriate removal rate, the initial Cr(VI) concentration of 50 mg/L was used for subsequent experiments.

#### 3.1.3. Co-existing metal ions

**Figure 3:**
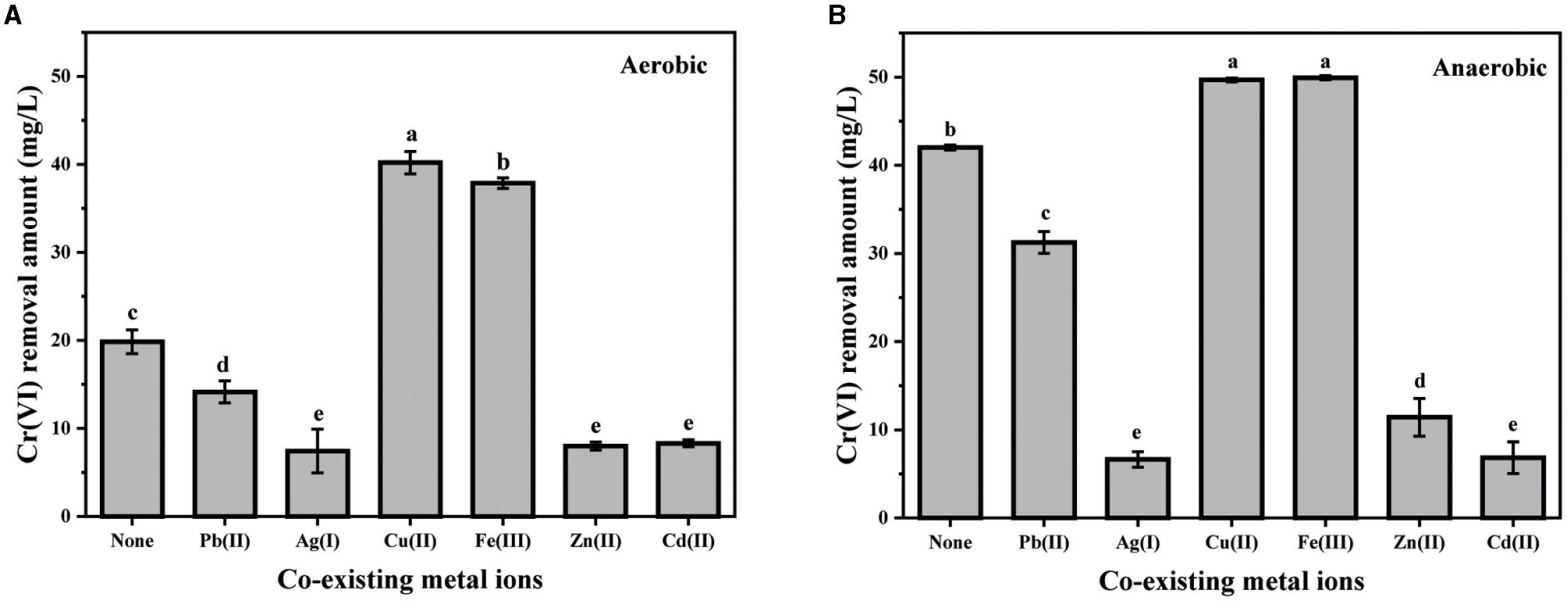
The effect of co-existing metal ions (25 mg/L) under aerobic **(A)** and anaerobic **(B)** conditions.

Considering that various heavy metal ions are usually co-existing in natural environments, the effects of several common metal ions, including Pb(II), Ag(I), Cu(II), Fe(III), Zn(II), and Cd(II), on the bacterial removal of Cr(VI) were then analyzed (Figure 3). Although their impact degrees were different, the influence trend of any certain kind of co-existing metal ions was coincidental under both aerobic and anaerobic conditions. Evidently, the co-existence of either Cu(II) or Fe(III) greatly enhanced the Cr(VI) removal capacity of PY14. Almost a complete removal of Cr(VI) was observed in the group with the addition of either Cu(II) or Fe(III), while only approximately 82% of Cr(VI) was removed in control under anaerobic conditions. Similarly, the Cr(VI) removal amount increased by approximately twice after the addition of either Cu(II) or Fe(III) under aerobic conditions. As reported, Cu(II) can be used as a prosthetic group for various enzymes (e.g., reductases) and is an essential component in the electron transport chain (Huang et al., 2021b). For example, Cu(II) was proven to nonspecifically bind chromate reductase NfoR from *Staphylococcus aureus* sp. LZ-01, leading to improved Cr(VI) reduction (Han et al., 2021). Moreover, Cu(II) can help the survival of bacteria by increasing the activity of antioxidant enzymes (Chen et al., 2021). The invigorating effect of Fe(III) was suggested by the production of a Fe(III)/F(II) redox couple by PY14 (an iron-reducing bacteria demonstrated in our previous study), which accelerated the electron transfer for Cr(VI) reduction (Huang et al., 2021a). On the contrary, Pb(II), Zn(II), Cd(II), and Ag(I) significantly inhibited the ability of this strain to remove Cr(VI), and the suppression became stronger successively. The inhibition of these heavy metal ions has also been reported in many other Cr(VI)-reducing bacteria (Banerjee et al., 2017; Zhu et al., 2019; Chen et al., 2021; Das et al., 2021; Huang et al., 2021b) because they either disrupted reductase activity or interfered with DNA repair and metabolic pathways.

#### 3.1.4. Organic electron shuttles

**Figure 4:**
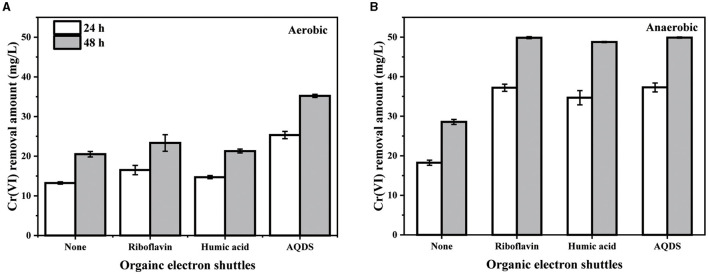
The effect of different organic electron shuttles at a final concentration of 10 μM under aerobic **(A)** and anaerobic **(B)** conditions.

Given the proven extracellularly respiratory characteristic of PY14 (Tang et al., 2023), several common organics with electron-shuttling functions, including riboflavin, humic acid, and AQDS, were selected to investigate their effect on Cr(VI) removal. Compared with the group using PY14 alone, the group supplemented with AQDS at a final concentration of 10 μM achieved a distinguishable improvement in the Cr(VI) removal capacity under aerobic conditions (Figure 4A). Meanwhile, the enhancement effect of adding either riboflavin or humic acid was relatively slight, which might imply that the strain had a preference for different electron shuttles under a certain condition (Rahman and Thomas, 2021). However, it was noteworthy that the addition of these three electron shuttles significantly improved the removal capacity of this strain under anaerobic conditions (Figure 4B). Particularly, in the presence of these electron shuttles, PY14 was able to remove almost 50 mg/L of Cr(VI) within 48 h, compared with 57% when it was alone. The observation that electron shuttles significantly promoted the bacterial removal of Cr(VI), especially under anaerobic conditions, has been reported in diverse Cr(VI)-reducing bacteria, such as *Escherichia coli* (Guo et al., 2012), *Shewanella oneidensis* (Meng et al., 2018), *Geobacter sulfurreducens* (He et al., 2019), *Aeromonas hydrophila* (Huang et al., 2019), *Cellulomonas* sp. (Field et al., 2013), and *Ochrobactrum intermedium* (Kavita and Keharia, 2012), although the degree of enhancement was largely dependent on bacterial species and electron shuttle type. Therefore, the electron-shuttling organics existing in natural environments are expected to *in situ* enhance the ability of PY14 to remediate Cr(VI) pollution.

### 3.2. Distribution and speciation of Cr(VI) reduction products

**Figure 5:**
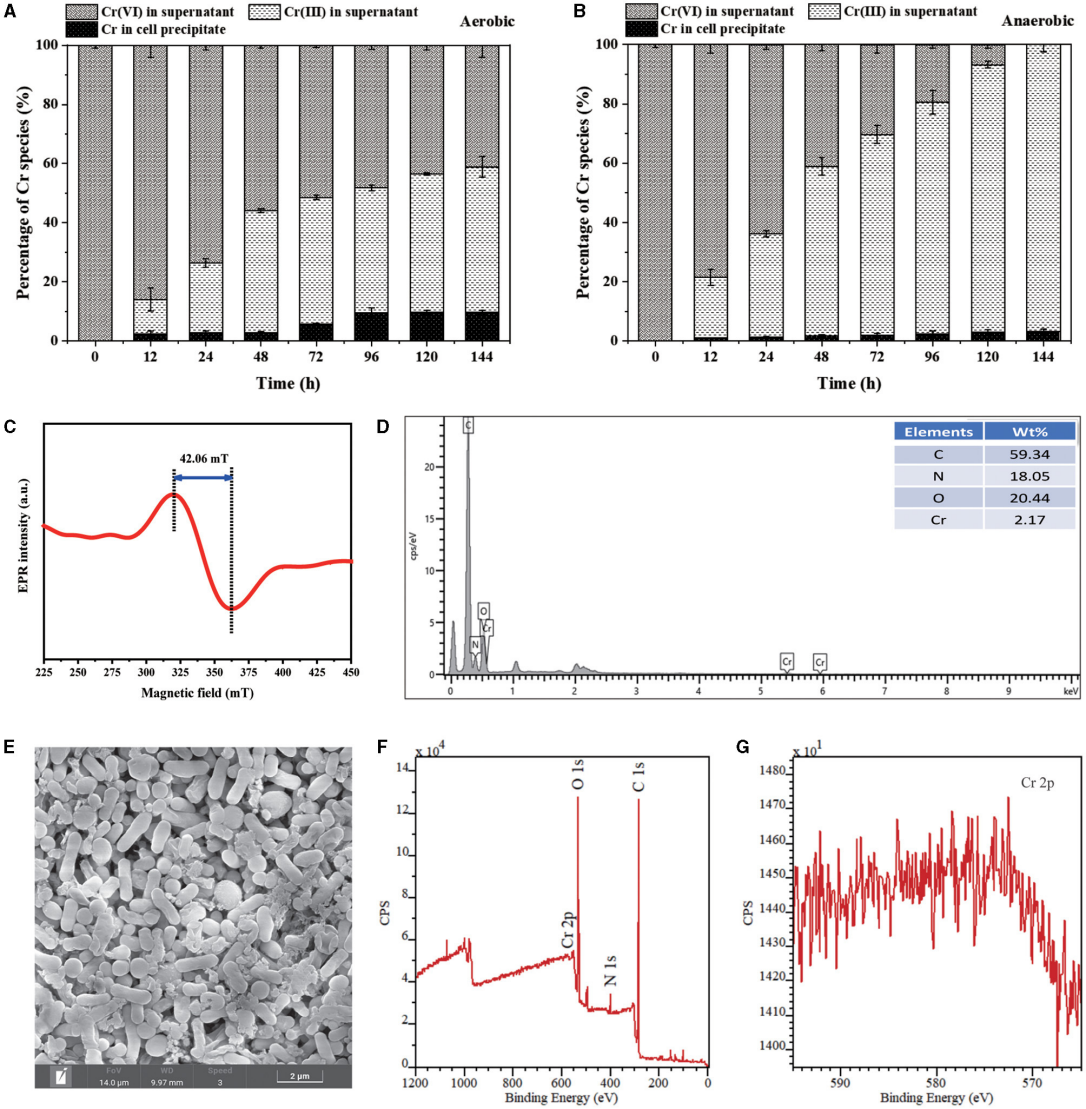
Characterization of Cr(VI) reduction products: the relative content of Cr in different culture fractions under **(A)** aerobic and **(B)** anaerobic conditions, **(C)** EPR spectrum of culture supernatant, **(D)** EDS spectrum, **(E)** SEM image, **(F)** XPS survey spectrum and **(G)** Cr 2p spectrum of cell precipitates.

The distribution and speciation of Cr during the removal process were investigated. Under both aerobic and anaerobic conditions, the proportion of Cr(VI) in the culture supernatant decreased gradually with reaction time, while that of Cr(III) increased significantly (Figures 5A, B). The result substantially proved that the reduction of Cr(VI) into Cr(III) by PY14 in the aqueous medium greatly promoted the toxic Cr(VI) removal. The EPR spectrum of culture supernatant after 144 h of reaction showed a broad peak at a g-factor of 1.93 with a magnetic field distance of 42.06 mT (Figure 5C), which was consistent with the characteristic peak of soluble organo-Cr(III) complexes reported previously (Puzon et al., 2005; Huang et al., 2019). On the contrary, the percentage of Cr existing in the cell precipitates under aerobic and anaerobic conditions was approximately only 9 and 3%, respectively, after 144 h of reaction. It was positively correlated with the density of bacterial cells under the two conditions and indicated that the biosorption by bacterial cells contributed little to the Cr(VI) removal. The low Cr content related to cell precipitates was also found for other reported Gram-positive bacteria that typically have a compact and simple cell wall, such as *Lysinibacillus* sp. HST-98 (Chen et al., 2021). Meanwhile, the EDS analysis (Figure 5D) also showed that the content of Cr element on the surface of PY14 cells was very low, although the element mapping image exhibited a uniform distribution of Cr element in the cell precipitates (Supplementary Figure S3). In addition, the SEM image (Figure 5E) showed the ellipsoidal or short rod-shaped cells of PY14, which was in agreement with our previous finding that the young cells of this train were rod-shaped and the old cells were nearly spherical (Tang et al., 2023). Then, the collected cell precipitates were analyzed by XPS after vacuum freeze-drying. The XPS spectra (Figure 5F) showed a weak signal of Cr 2p at a binding energy of 575 eV, which was too weak to be used to distinguish its chemical valence state (Figure 5G), further indicating a very small amount of Cr element existing in the cell precipitates. Taken together, these results suggested that organo-Cr(III) complexes were the dominating final product of Cr(VI) reduction by PY14. The formation of organo-Cr(III) complexes with less toxicity during bacterial Cr(VI) reduction was common (Li et al., 2008; Dogan et al., 2011; Huang et al., 2019, 2021a), especially in small-molecule organic-rich matrixes such as LB medium, which could be an important survival strategy for bacteria in Cr(VI)-contaminated environments.

### 3.3. Contribution of cell components to Cr(VI) reduction

**Figure 6:**
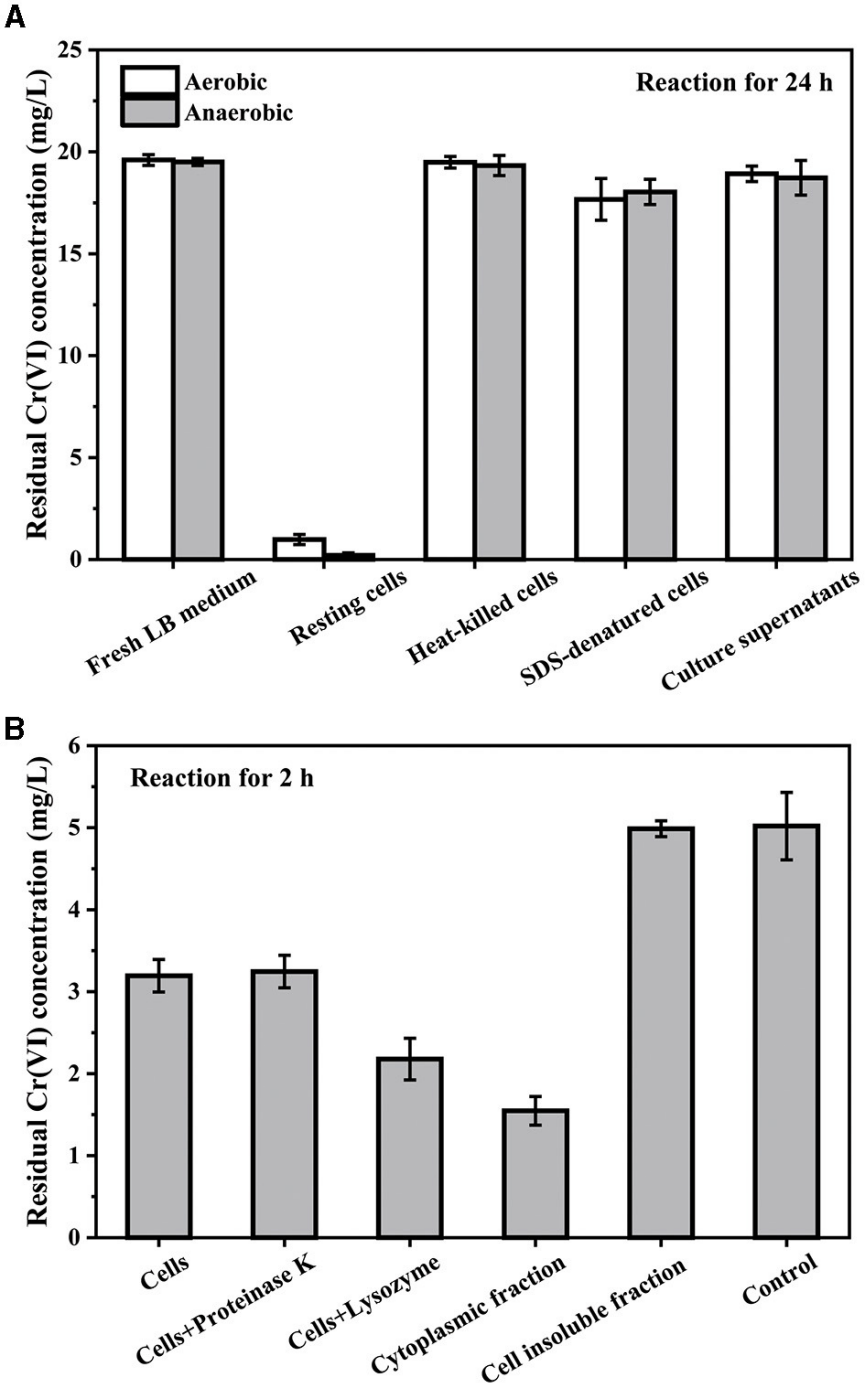
The residual Cr(VI) concentration after reaction with different cell components: initial Cr(VI) concentration of 20 mg/L in LB medium **(A)** and 5 mg/L in PBS buffer **(B)**, respectively.

To determine the mechanism of Cr(VI) reduction by PY14, the contributions of culture supernatant, vigorous, and denatured cells were compared. After the bacterial cells were cultured to the logarithmic growth stage, the bacterial cells were collected by centrifugation as resting cells. As shown in Figure 6A, the resting cells resuspended in fresh LB medium of equal volume removed more than 95% of Cr(VI) at an initial concentration of 20 mg/L within 24 h under both aerobic and anaerobic conditions, and yet, the fresh LB medium and the culture supernatant had little removal capacity. In addition, after heat killing and SDS denaturation, the resting cells almost lost their ability to remove Cr(VI). This result indicated that the reduction of Cr(VI) was mainly attributed to the cells rather than the culture supernatant, although it has been previously confirmed that the reduction products existed in the form of organo-Cr(III) complexes in the culture supernatant. This further excluded cell adsorption as a significant approach for Cr(VI) removal. Then, the abilities of different cell components, including cytoplasm and cell membrane proteins, in Cr(VI) reduction were compared in 0.01M PBS buffer with the addition of 1 mM NADH as electron donors (Figure 6B). Expectedly, the resting cells showed a distinct ability to reduce Cr(VI) at an intimal concentration of 5 mg/L in comparison to the control. After the digestion of out-membrane proteins by proteinase K (Long et al., 2022), the cells retained a similar Cr(VI)-reducing capability, suggesting the ignorable effect of out-membrane proteins on Cr(VI) reduction. However, after treatment with lysozyme, the Cr(VI)-reducing capability increased significantly, indicating that the leakage of the cytoplasm could promote the reductive removal of Cr(VI).

Furthermore, after ultrasonic fragmentation of the resting cells, the cytoplasmic fraction was collected through centrifugation for the Cr(VI)-reducing test. It was found that the cytoplasmic fraction exhibited a much higher capacity for Cr(VI) reduction than the resting cells, indicating the significant contribution of the cytoplasmic fraction to Cr(VI) reduction. However, the residual cell insoluble fraction had almost no Cr(VI)-reducing ability (Figure 6B), which further suggested that the cell membrane components were not involved in Cr(VI) reduction. This view that the cytoplasm was the primary site for Cr(VI) reduction was also supported by previous literature on other strains of *Exiguobacterium* species (Alam and Malik, 2008; Das et al., 2021). Taken together, intracellular reductases, rather than extracellular or membrane-bound reductases, were the main participants in the biotransformation of highly toxic Cr(VI) into less toxic Cr(III).

### 3.4. Genes related to Cr(VI) metabolism

The draft genome of strain PY14 was sequenced to determine the possible genes related to Cr(VI) metabolism. The results showed that the estimated genome size was 2,997,078 base pairs (bp), with a G+C content of 51.58%. The reads were assembled into 50 contigs and 33 scaffolds, with a sequencing coverage of 473 fold. The N_50_ length of the contigs was 339,536 bp, and 3,092 CDSs were predicted in the draft genome sequences. The CDSs were used for homology analysis and annotation with the NR, KEGG, COG, and Swiss-Prot databases; the genes with chromate reduction and transport functions are shown in Table 1. Two putative genes, *chrA* and *chrR*, were annotated to encode Chr family proteins that were reported to be involved in bacterial chromate metabolism. Protein ChrA was identified as a transporter responsible for chromate efflux in *P. aeruginosa* and *Cupriavidus metallidurans* (Ramírez-Díaz et al., 2008), which endowed the bacteria with chromate resistance. Owing to the structural similarity between chromate oxyanion and sulfate oxyanion, the sulfate transporter was considered the potential carrier responsible for the entry of chromate into bacterial cells (Salnikow and Zhitkovich, 2008). Two genes (accession: MDL5376491.1 and MDL5376674.1) are annotated as sulfate transporter coding genes, which might take charge of the chromatin influx into PY14 cells. Since ChrR was identified as a Class I chromate reductase in several bacteria, such as *P. putida* (Park et al., 2000; Ackerley et al., 2004b), the existence of the *chrR* gene in the genome of PY14 suggested its contribution to chromate reduction. The gene annotated as *nfrA1*, belonging to *nfsA*, encodes oxygen-insensitive NADPH nitroreductase. NfsA was previously reported as a chromate reducer in *E. coli* (Ackerley et al., 2004a). In addition, three other genes with accession numbers MDL5375555.1, MDL5376943.1, and MDL5377576.1, respectively, were also annotated to encode nitroreductase family proteins, though it was unclear whether they were involved in chromium reduction in PY14. The gene *acpD* encoding FMN-dependent NADH-azoreductase was also identified in the genome of PY14, which showed a high identity to AzoR (another chromate reductase found in *E. coli*) (Robins et al., 2013). These results demonstrated the existence of multiple putative chromate reductases and transporters in strain PY14, indicating its versatile intracellular Cr(VI) reduction mechanism.

**Table 1:** Putative genes related to Cr(VI) metabolism in the genome of strain PY14.

**Gene accession**	**Putative name**	**Predictive function**	**Identities**
MDL5376476.1	*chrA*	Chromate efflux transporter; chromate transporter (*Exiguobacterium alkaliphilum*)	365/394 (93%)
MDL5375720.1	*chrR*	Chromate reductase, NADPH-dependent FMN reductase (*Exiguobacterium* sp. AT1b)	153/185 (83%)
MDL5376491.1	*-*	SulP family inorganic anion transporter (*Exiguobacterium alkaliphilum*)	465/493 (94%)
MDL5376674.1	*-*	SulP family inorganic anion transporter (*Exiguobacterium* sp. AT1b)	440/514 (86%)
MDL5375493.1	*nfrA1*	Oxygen-insensitive NADPH nitroreductase (*Exiguobacterium alkaliphilum*)	212/249 (85%)
MDL5377051.1	*acpD*	FMN-dependent NADH-azoreductase; NAD(P)H-dependent oxidoreductase (*Exiguobacterium alkaliphilum*)	197/211 (93%)

## 4. Conclusion

In summary, several significant factors that influence the reductive removal of aqueous Cr(VI) by a Gram-positive facultative anaerobic *Exiguobacterium* sp. PY14, and the reduction mechanism were elucidated under both aerobic and anaerobic conditions. This strain, PY14, showed the strongest Cr(VI) removal ability at a moderately alkaline pH of 8.0 because of its basophilic growth. The increase in the initial concentration of Cr(VI) depressed the removal percentage and rate due to its biotoxicity to PY14 cells. The removal rate constant for 50 mg/L of Cr(VI) under anaerobic conditions (1.82 × 10^−2^ h^−1^) was 3.3 times that under aerobic conditions, proving that PY14 had superior Cr(VI) removal ability under the former condition. The co-existence of Fe(III) and Cu(II) significantly promoted the removal of Cr(VI), while the effects of Ag(I), Pb(II), Zn(II), and Cd(II) were quite contrary. In addition, electron-shuttling organics, such as riboflavin, humic acid, and AQDS, promoted the Cr(VI) removal to varying degrees, and the effect was more significant under anaerobic conditions. By analyzing the contribution of different cell components and the speciation of products, the removal of aqueous Cr(VI) by PY14 was demonstrated to be attributed to the reduction in the cytoplasm rather than outside the cells. However, the end products existed in the aqueous solution in the form of an organic-Cr(III) complexes. Several possible genes involved in Cr(VI) metabolism were identified through genomic sequencing, alignment, and annotation. Particularly, the genes (*chrR* and *chrA*) that encode well-known Chr family proteins responsible for chromate reduction and transport existed in the genome of PY14. Several genes also encode nitroreductase, azoreductase, and sulfate transporters, which have also been documented to be involved in Cr(VI) metabolism. The progress of this study in the roles of crucial influencing factors and reduction mechanisms will be conducive to promoting the potential application of *Exiguobacterium* sp. PY14 with high adaptability to environmental stress in the removal of Cr(VI) contamination in the actual environment.

## Data availability statement

The datasets presented in this study can be found in online repositories. The names of the repository/repositories and accession number(s) can be found in the article/Supplementary material.

## Author contributions

YH: formal analysis, study design, data acquisition, funding acquisition, and writing–original draft. JT: study design, method development, data acquisition, and writing–editing. BZ: formal analysis, data acquisition, and writing editing. Z-EL and HN: study design, method development, and writing–editing. XF: study design and writing–editing. LZ: study design, data acquisition, funding acquisition, project management, supervision, and writing–drafting and editing. All authors contributed to the article and approved the submission.
