# Supplementary material for: Influencing factors and mechanism of Cr(VI) reduction by facultative anaerobic Exiguobacterium sp. PY14
Source: Front Microbiol. 2023 Aug 10;14:1242410. doi: 10.3389/fmicb.2023.1242410 (PMC10449125; doi:10.3389/fmicb.2023.1242410)
Supplement: Supplementary file 1 [file Data_Sheet_1.PDF]

*Supplementary Material*

# Factors affecting Cr(VI) removal by a facultative anaerobic *Exiguobacterium* sp. PY14 and its reduction mechanism

**Yunhong Huang[1], Jie Tang[1], Bei Zhang[2], Zhong-er Long[1], Haiyan Ni[1], Xueqin Fu[1], Long Zou[1]***

[1]Nanchang Key Laboratory of Microbial Resources Exploitation & Utilization from Poyang Lake Wetland, College of Life Sciences, Jiangxi Normal University, Nanchang, 330022, China

[2]College of Art and Design, Jiangxi Institute of Fashion Technology, Nanchang, 330201, China

***Correspondence:**
Long Zou
zoulong@jxnu.edu.cn

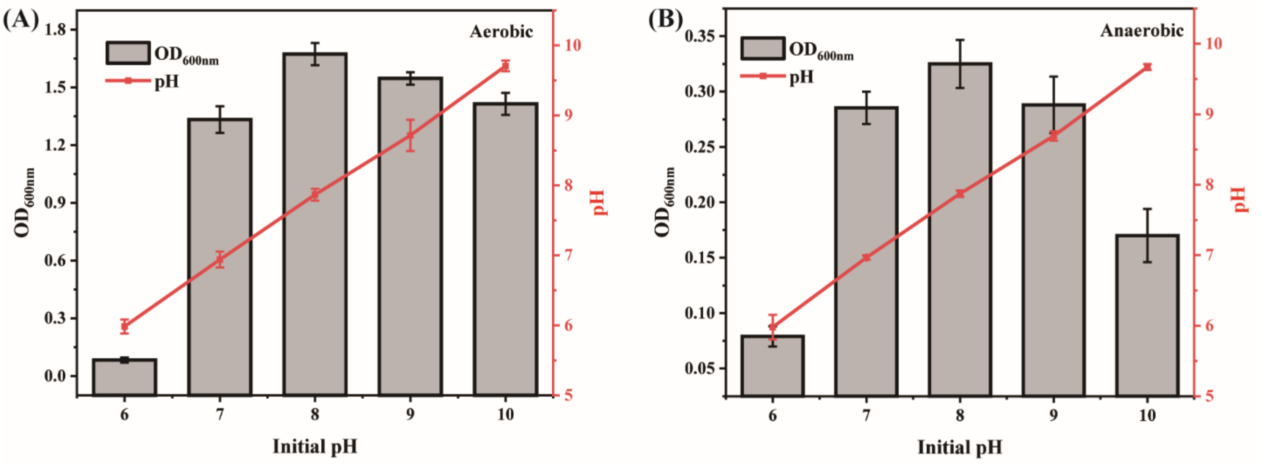


**Fig. S1** The bacterial cell density (OD600nm) and pH value after 96 h of reaction under aerobic (A) and anaerobic (B) conditions.

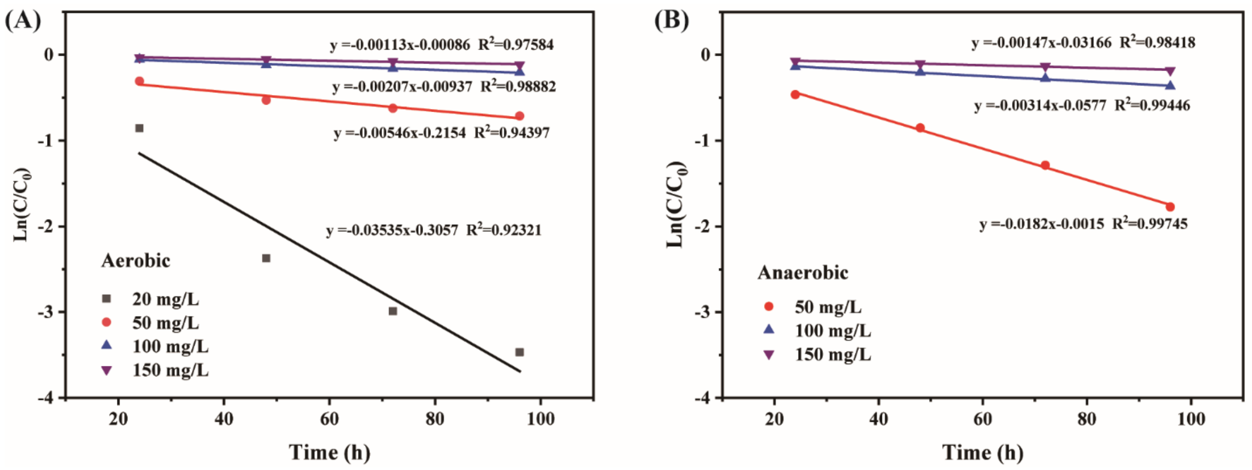


**Fig. S2** The kinetics of Cr(VI) removal for different initial Cr(VI) concentrations under aerobic (A) and anaerobic (B) conditions.

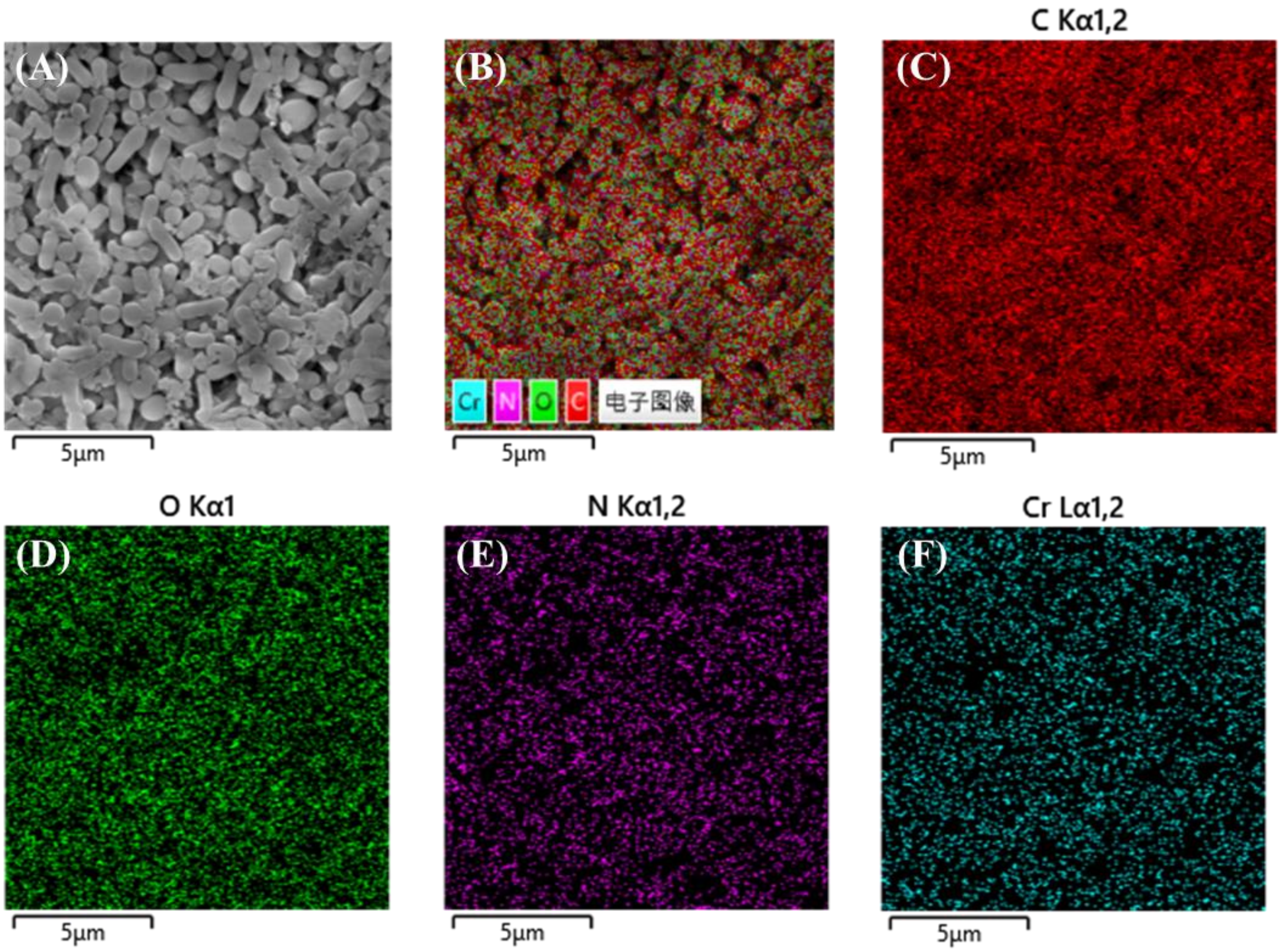


**Fig. S3** EDS elemental mapping analysis of cell precipitates.
